# Explainability and causability in digital pathology

**DOI:** 10.1002/cjp2.322

**Published:** 2023-04-12

**Authors:** Markus Plass, Michaela Kargl, Tim‐Rasmus Kiehl, Peter Regitnig, Christian Geißler, Theodore Evans, Norman Zerbe, Rita Carvalho, Andreas Holzinger, Heimo Müller

**Affiliations:** ^1^ Diagnostic and Research Institute of Pathology Medical University of Graz Graz Austria; ^2^ Charité‐Universitätsmedizin Berlin, Corporate Member of Freie Universität Berlin and Humboldt‐Universität zu Berlin Institute of Pathology Berlin Germany; ^3^ DAI‐Labor, Agent Oriented Technologies (AOT) Technische Universität Berlin Berlin Germany; ^4^ Human‐Centered AI Lab University of Natural Resources and Life Sciences Vienna Vienna Austria

**Keywords:** digital pathology, artificial intelligence, explainability, causability

## Abstract

The current move towards digital pathology enables pathologists to use artificial intelligence (AI)‐based computer programmes for the advanced analysis of whole slide images. However, currently, the best‐performing AI algorithms for image analysis are deemed black boxes since it remains – even to their developers – often unclear why the algorithm delivered a particular result. Especially in medicine, a better understanding of algorithmic decisions is essential to avoid mistakes and adverse effects on patients. This review article aims to provide medical experts with insights on the issue of explainability in digital pathology. A short introduction to the relevant underlying core concepts of machine learning shall nurture the reader's understanding of why explainability is a specific issue in this field. Addressing this issue of explainability, the rapidly evolving research field of explainable AI (XAI) has developed many techniques and methods to make black‐box machine‐learning systems more transparent. These XAI methods are a first step towards making black‐box AI systems understandable by humans. However, we argue that an explanation interface must complement these explainable models to make their results useful to human stakeholders and achieve a high level of causability, i.e. a high level of causal understanding by the user. This is especially relevant in the medical field since explainability and causability play a crucial role also for compliance with regulatory requirements. We conclude by promoting the need for novel user interfaces for AI applications in pathology, which enable contextual understanding and allow the medical expert to ask interactive ‘what‐if’‐questions. In pathology, such user interfaces will not only be important to achieve a high level of causability. They will also be crucial for keeping the human‐in‐the‐loop and bringing medical experts' experience and conceptual knowledge to AI processes.

## Introduction

During the last decade, technological advancements in whole slide images (WSIs) and approval for clinical use by regulatory agencies in many countries have paved the way for implementing digital workflows in diagnostic pathology. This shift to digitisation enables pathologists to view, examine and annotate histopathological slides seamlessly in various magnifications on a computer screen and facilitates telepathology and obtaining a second opinion from (remote) colleagues. However, digital pathology is not just a modern version of the conventional microscope but the foundation for computational pathology. It enables the usage of artificial intelligence (AI) to aggregate information from multiple sources of patient information including WSIs. Thus, this big‐data approach to pathology opens up entirely new capabilities and will change the pathology practice [1, 2].

Over the last few years, many computational approaches for the advanced analysis of WSIs have been developed. These applications aim to support pathologists with tedious routine tasks, improve the accuracy of diagnosis, and aid in exploring new diagnostic and prognostic criteria in many pathology subspecialties, including breast pathology [3], lung pathology [4], prostate [5, 6], musculoskeletal [7], and dermatopathology [8]. Many of these computational approaches deliver results comparable to human experts or may even outperform human pathologists in specific tasks [9]. Furthermore, computational WSI analysis enables exploring aspects beyond human capabilities using ‘sub‐visual’ information. An example is the prediction of molecular genetic alterations from histopathologic morphology [10]. Most of these applications utilise AI, specifically machine learning (ML) techniques to achieve such promising results. However, these AI applications have become increasingly opaque, meaning that it is often impossible to retrace and understand how a result was generated. Therefore, there is a growing call for explainability, especially in high‐stakes domains such as medicine.

This review aims to shed light on the issue of explainability in digital pathology:

1. To provide insights into the context of explainability in digital pathology, we briefly dive into the basic principles of ML.
2. We explain the notion of explainability and give an overview of the state‐of‐the‐art in relevant explainability techniques.
3. We introduce the concept of causability and describe why explainability and causability are particularly important in pathology.
4. We discuss current research strands and open questions in this field.

## ML: basic principles

The concept of explainability in digital pathology is closely linked to ML applications in this domain. To lay the ground for the following sections, we briefly introduce basic principles and limitations of ML without going into technical details.

ML, a subfield of AI, comprises computer programmes that can automatically learn and improve from ‘experience’ without following explicit instructions. This distinguishes ML from traditional software engineering, where the developers explicitly define and encode the rules to determine how the software handles the input data for a specific output. In ML, the algorithm approximates input–output relations from data using numerical optimisation and statistics. Most ML approaches define a solution space (also called ‘model’), a loss function and a learning algorithm that searches for solutions that minimise the loss function. In unsupervised learning, a sub‐discipline of ML, the algorithm attempts to find patterns in the input data. Unsupervised ML is used for clustering and automatically recovering structure in data or feature extraction for other ML types. In supervised learning, the algorithm receives a so‐called training dataset, which includes samples of input data and corresponding output data (often called labels). The ML algorithm autonomously derives a model that best describes input–output relations for these training data. The goal is to train an ML model which does not only perform well on the training data but can also make highly accurate predictions for previously unseen data – an ability called generalisation. A test dataset, not previously seen by the model, is used to test the generalisation‐ability of the ML model.

Current ML applications for classification tasks in pathology, where the algorithm predicts a discrete class (e.g. tumour/non‐tumour) and for regression tasks, where the algorithm predicts a continuous value (e.g. likelihood of tumour metastasis), are mostly based on training with labelled data [11]. Unsupervised ML techniques are mainly used for assisting tasks along the ML pipeline. For example, clustering can be used to propagate labels from a subset of annotated data to remaining unlabelled data [12].

Generally, the ML, model after training on images, is a complex high‐dimensional non‐linear mathematical function that can become quite complicated, depending on the complexity of the task and the ML algorithms used. For example, deep learning (DL) algorithms, a family of ML algorithms using ‘deep’ (i.e. multi‐layered) neural networks, yield ML models comprising millions of parameters and are so complex that humans cannot understand relationships amongst variables to form the model's prediction. Such ML models are called ‘black‐box’ models [13].

An ML model, which reaches high prediction accuracy for the test dataset, can still show decreased prediction accuracy in a real‐world setting. Two common reasons for prediction failures of ML models are out‐of‐distribution (OOD) data and spurious correlations.

### Out‐of‐distribution data

The prediction performance of an ML model is approximated under the assumption of being applied to identically distributed data as encountered during the training. For OOD data, the performance may be substantially different. An illustrative example for such OOD input data would be to present an immunohistochemistry (IHC) image to an ML model that was trained solely with haematoxylin and eosin stained slides. However, especially in digital pathology, issues with OOD data are often more subtle. For example, staining variations between pathology laboratories can result in decreased performance of an ML application when trained on WSIs of a single laboratory and applied to WSIs of another laboratory [14].

### Spurious correlations

The predictions of an ML model are based on the input–output relations it has learned from the training data. However, the input–output relations learned by the ML model are not causal relationships but only correlations. An ML model may also base its predictions on spurious correlations, i.e. ones that occur purely by chance. Confounding variables, which cause spurious correlations, may be artefacts visible to humans. For example, predictions of an ML model for the recognition of melanoma in dermoscopic images have shown associations with surgical skin markings [15]. However, confounding variables in digital pathology may also be hidden variables such as patient age, preparation date or origin of histology slide, or WSI scanner [16].

In practice, such decreased prediction accuracy and prediction failures of ML models are a problem if they cannot be identified and recognised by the human who relies on the machine's decision. Users must be aware of the limitations of AI applications and consider their potential flaws [17]. Therefore, if an AI system is supposed to contribute to a medical decision, where wrong decisions can have significant adverse effects on patients, medical experts must have the means to retrace and understand machine decision processes [13]. The lack of explainability of black‐box models is often named as a significant obstacle to introducing these applications in high‐stakes areas such as medicine [18].

## Explainability and approaches of explainable AI

‘Explainability’ refers to a characteristic of an AI system which enables humans to understand why the AI system delivered the presented predictions [17]. Technically speaking, explainability highlights those elements of input data and ML model which contributed to the AI system's output [19].

It can be differentiated into global explanations, which aim to provide insights into the inner workings of the ML model and make the model transparent as a whole, and local explanations, which aim to explain a specific prediction of the ML model [20, 21]. Furthermore, two types of AI systems can be distinguished concerning the time when explainability is obtained: ante hoc explainable systems and post hoc explainable systems [22]. Ante hoc explainable systems are inherently interpretable by design since they use ML algorithms that generate models which are directly human‐interpretable. Classical examples include decision trees, linear regression and fuzzy inference systems [23], but there are also approaches to create interpretable DL algorithms [24, 25]. Post hoc explanation methods generate explanations for so‐called black‐box ML models, which are too complex to be directly interpretable. Post hoc explanation methods usually do not aim to explain how an ML model functions, but they aim to explain why it made a specific prediction [21].

During the last decade, the research topic of explainable AI (XAI), a subfield of ML, has developed many methods and mechanisms to provide human‐interpretable explanations for complex ML models. Several review papers (e.g. [25, 26, 27]) provide detailed overviews of current XAI techniques.

It can be distinguished between XAI methods aiming to explain the inner workings of the ML model, XAI methods seeking to explain a specific prediction of the ML model and XAI methods aiming to estimate the uncertainty of the ML model's prediction [25]. So‐called model‐ground explanations, i.e. XAI methods that aim to explain the inner workings of the ML model, are mainly targeted at the developers of the ML system who want to gain insights on how to improve the system [28]. In contrast, explanations of the ML model's predictions and uncertainty are highly relevant to the users of the ML application.

**Figure 1:**
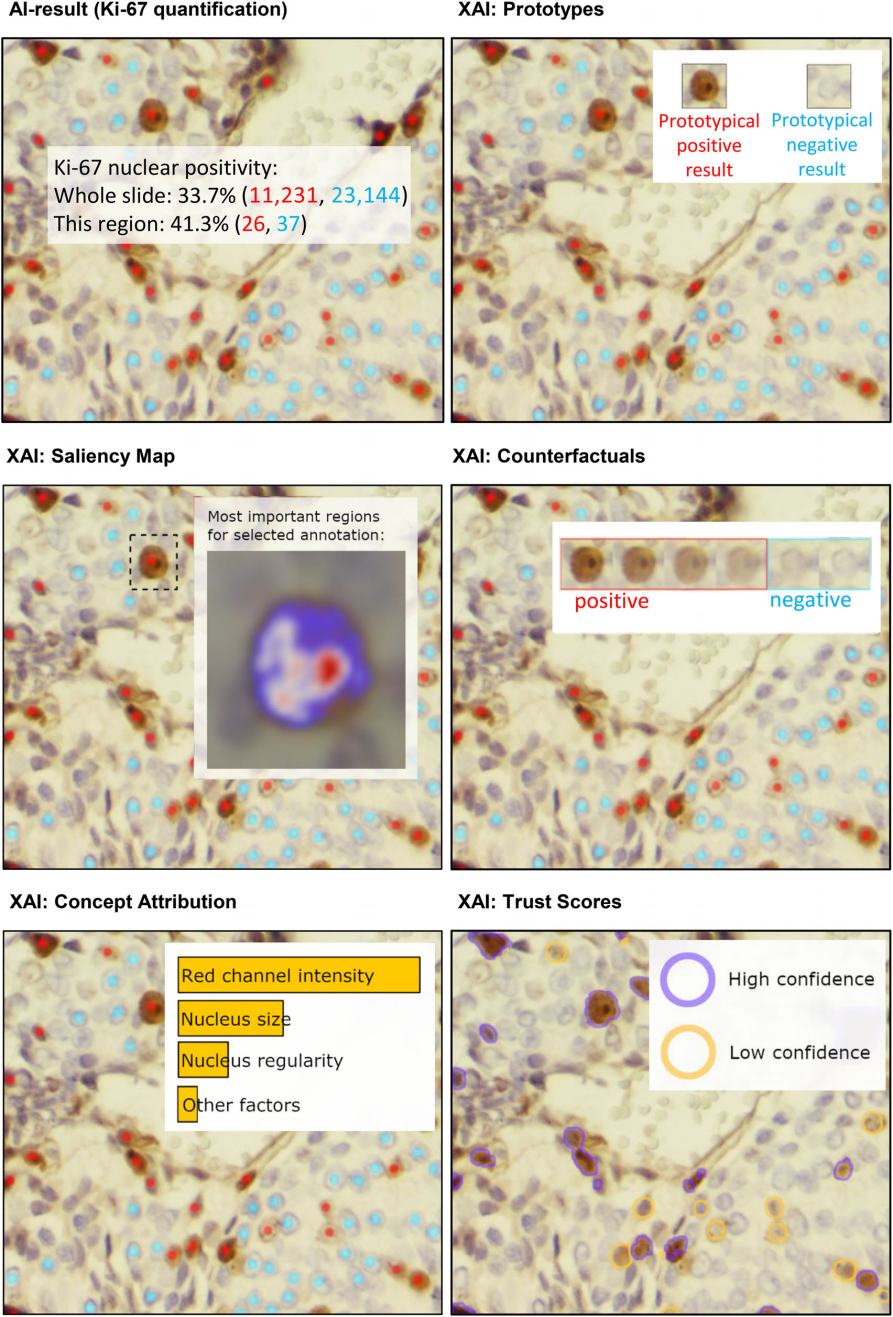
Popular presentation modalities of XAI methods are prototypes (show representative input examples for the predicted classes), counterfactuals (show how minimal changes in the input would lead to contrastive output), trust scores (highlight areas where the ML model's uncertainty is high), saliency map (visualises estimated relevance of input pixels to the ML model's output) and concept attribution (depict the estimated relevance of human‐defined concepts to the ML model's prediction). Here these presentation modalities of XAI methods are exemplified through AI results for Ki‐67 quantification (graphic adapted from [29]).

In pathology, where typical tasks for ML applications are related to WSI analysis, explanations are best conveyed to the user by visualization on the input image or synthetic visualisations [29]. Popular presentation modalities of XAI methods in the imaging domain include saliency maps, concept attribution, prototypes, counterfactuals, and trust scores (see Figure 1) [29, 30].

*Saliency maps* show the estimated relevance of each (group of) pixel(s) of the input image to the ML model's prediction in the form of a visual heatmap overlay on the input image. Various techniques are available to estimate the relevance of input pixels to the ML model's prediction. Often, these techniques result in divergent explanations for the same input image [30, 31]. For non‐experts in the XAI field, who often do not have sufficient knowledge and information to understand which technique has been applied to create a specific saliency map and what the limitations of that specific approximation technique are, saliency maps might provide a misleading explanation. Thus, although they are one of the most widespread XAI methods, saliency maps should be applied and interpreted with caution [21].

*Concept attribution* techniques depict the estimated relevance of human‐defined concepts (e.g. colour, area, architecture of nuclear features, mitosis) to the ML model's prediction in the form of relevance scores [32].

*Prototypes* are a so‐called ‘explanation by example’ technique. The prototypes method aims to convey the common features of a specific prediction class by showing representative input examples for the respective prediction [30]. Prototypes can either be examples picked from the model's training dataset or synthetic examples [33, 34].

*Counterfactuals* are also an ‘explanation by example’ technique. The counterfactuals method displays examples of input data, which are similar to the specific input but result in a different output of the ML model. Counterfactuals aim to reveal necessary minimal changes in the input so that one would obtain a contrastive output of the ML model [30, 35].

*Trust scores* provide estimates for the ML model's uncertainty and help to better understand the limitations of the ML model resulting from the model's training data and training scheme (i.e. reducible [epistemic] uncertainty) as well as the limitations resulting from the fact that any ML model is only a simplified emulation of the real world (i.e. irreducible [aleatoric] uncertainty) [25]. Uncertainty measures can be visualised as overlays on the input image to highlight areas where the ML model's uncertainty is high [25]. Alternatively, they can be used to calculate confidence intervals of the model's output [36].

Since post hoc explanations created by XAI techniques are simplified approximations of complex ML models, it is vital to be aware of their limitations. Users must be informed where, i.e. for which domain and task, the underlying approximations are valid and reliable and where such an explanation might become inaccurate and misleading [21]. In addition to the risk that the recipient of a post hoc explanation is not aware of its limitations, there is also a risk that (ambiguous) post hoc explanations unintentionally support positive confirmation bias and, thus, false interpretations by the recipients [29]. Furthermore, post hoc explanations can be intentionally misused to foster inadequate trust, for example, by falsely attributing an AI decision to an irrelevant but acceptable feature [21, 37].

## From explainability to causability

AI applications in high‐stakes domains such as pathology must perform well and reliably. In addition, they should also be interpretable and explainable for a human expert. Indeed, various stakeholder groups in pathology have different requirements regarding explainability [38]: in clinical use, explainability should enable the pathologist to make an informed decision on whether or not to rely on the system's recommendations, specifically in cases where the AI system and the medical expert disagree [17]. Explainability should support the pathology laboratory's quality assurance in understanding the limitations of the AI application and the ML model's ability to generalise to the specific framework conditions of that laboratory [25]. Finally, medical researchers also require explainability, as they seek to uncover new insights from AI and thus need to understand the causality of patterns learned by an ML model [13]. However, current best‐performing AI approaches typically rely on statistical correlations and cannot build causal models to support human understanding. There is a need to disentangle correlation from causation to avoid providing misleading explanations [39].

**Figure 2:**
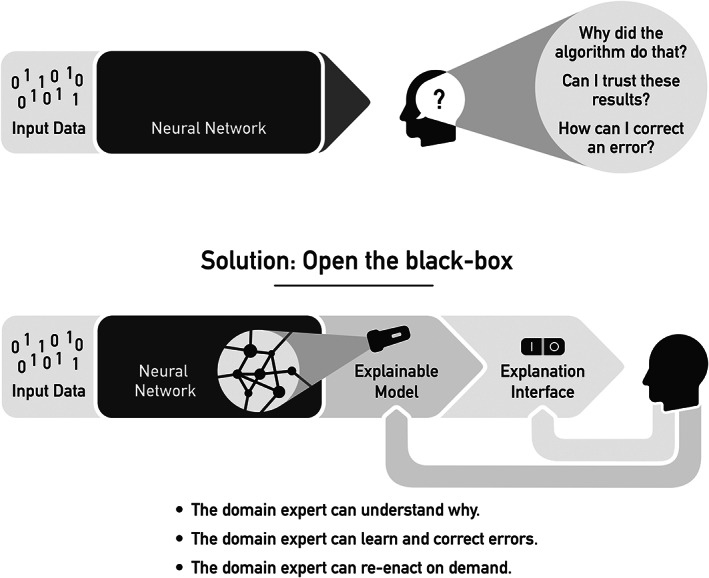
XAI techniques help to make black‐box ML models more transparent and explainable. However, these explainable models must be complemented by an explanation interface to deliver results that are useful to the users and achieve high causability.

As described in the previous section, XAI methods are a first step towards making black‐box models better understandable by humans. However, an explainable model (XAI) is not sufficient. To make the results gained by that model useful to the human stakeholder, an explanation interface must complement the explainable model (see Figure 2) [13, 40]. Ideally, as shown recently [29], the explanation interface for an AI system in digital pathology should be interactive, enabling the user to retrieve explanations on demand and to ask ‘why’ and ‘what‐if’ questions to refine the understanding of explanatory factors underlying the ML model's prediction. Holzinger *et al* [13] have coined the term ‘causability’ as a measure of the usability of such explanation interfaces:

*Causability is the extent to which an explanation of a statement to a human expert achieves a specified level of causal understanding with effectiveness, efficiency and satisfaction in a specified context of use*. [13]

Thus, similar to the well‐known term usability, which is widely utilised in human–computer interaction to measure the quality of use, causability measures the quality of explanations in human–AI interaction. As such, causability is critical for successfully designing, developing and evaluating human–AI interfaces [41]. In digital pathology, there is a need for effective and efficient human–AI interfaces to empower human experts to take responsibility for their AI‐supported decision‐making by helping them better understand the underlying factors for an ML model's prediction. In addition, human–AI interfaces should also enable humans to complement AI with conceptual knowledge, experience, and contextual understanding [42]. For example, the graphical user interface of an AI solution for automatic Ki‐67 quantification in digital pathology (as shown in Figure 1) should not only visualise in an overlay on the WSI all cells the AI has classified as K‐i67‐positive tumour cells, but should also enable the pathologist to easily provide corrective feedback, for example by clicking on cells misclassified by the AI. Moreover, human–AI interfaces play a crucial role in keeping the human always in control, which is essential in the medical field for social, ethical and legal reasons [41].

## Explainability and causability in the regulatory context

The regulatory landscape for market approval of medical devices is different across continents and countries and it is beyond the scope of this article to give a detailed and comprehensive overview. Instead, in the following paragraphs, the importance of explainability and causability in the regulatory context is illustrated based on the example of the European *in vitro* diagnostic medical devices regulation (IVDR).

In Europe, AI applications for WSI analysis in diagnostics are regarded as IVD as defined in article 2(2) of the European IVDR [43]. To apply for market approval of an IVD in the European Union, the IVDR requests manufacturers to provide evidence of scientific validity, analytical performance and clinical performance of the IVD.

### Role of explainability and causability in demonstrating scientific validity

To provide evidence for the scientific validity of an AI application, it must be demonstrated that there is a scientifically proven link between the output of the AI application and the targeted physiological state or clinical condition as defined in the intended purpose of the AI application. Demonstrating such an association of the output of the AI application with the targeted clinical condition or physiological state is impossible with a black‐box ML approach. Still, it requires explainability and causability to answer the question: *Why does the AI application work in general and generate reliable results for the intended purpose?* [44]. However, for demonstration of scientific validity, it must be distinguished between (1) an AI algorithm that supports or replaces an existing diagnostic method that scientific studies have already validated and (2) an AI algorithm that itself contributes significantly to the scientific and diagnostic approach [44].

An illustrative example for the first case would be an AI algorithm that assists pathologists with quantifying the Ki‐67 labelling index (i.e. the percentage of Ki‐67 IHC‐stained tumour nuclei), a well‐established indicator for cellular proliferation used for diagnosis and prognosis assessment in various cancers [45]. Manufacturers of the AI algorithm may refer to the existing scientific studies providing evidence for the scientific validity of Ki‐67 as a cellular proliferation index. However, in addition, the algorithm must demonstrate that its results (i.e. the Ki‐67‐values determined by the algorithm) are indeed based on the percentage of Ki‐67‐stained tumour cell nuclei of the input WSI data. To show this, the algorithm should: (1) mark the identified tumour cell nuclei as Ki‐67‐positive or ‐negative on the WSI and (2) compute the Ki‐67 result as the percentage of positive tumour cells in the region of interest. This visualisation of the algorithm's outcome makes the algorithm's prediction process retractable and verifiable by a medical expert.

An illustrative example for the second case, an AI algorithm that significantly contributes to the scientific and diagnostic approach, is a survival prediction algorithm for colorectal cancer which was not trained to identify already known features but used a weakly supervised learning approach to find novel features [46]. By analysing the algorithm's results, medical experts found that small groups of tumour cells in the vicinity of fat cells, called ‘tumour‐associated fat’ (TAF), constituted a feature of high‐risk clusters found by the algorithm. However, scientific studies have not yet validated the diagnostic approach based on TAF. Thus, to provide evidence for the scientific validity of the AI algorithm, it would be necessary to conduct independent validation studies to demonstrate the correlation of the presence of TAF with the survival of colorectal cancer patients in independent cohorts.

### Role of explainability and causability in analytical performance evaluation

Evaluation of the analytical performance should demonstrate the AI application's ability to reliably and accurately generate the intended analytical output from the input data over the whole range of the intended use [47]. Thus, it is especially important that test datasets used for the evaluation of the analytical performance of an AI application cover features of the entire intended purpose of the AI application. Such features include, amongst others, population and disease spectrum, specimen preparation and handling, preanalytical parameters (e.g. fixation, staining), scanning process (e.g. scanner type, resolution, compression, colour calibration) and WSI quality (e.g. stitching errors, air bubbles, and out‐of‐focus areas) [48].

XAI methods are important tools to consult when an AI application cannot deliver reliable results for a given input dataset. Specific reasons may be the low quality of input data or features of the input data which were not present in the training data. Furthermore, XAI methods should help to recognise so‐called ‘Clever Hans’ predictors and detect if the model learned any shortcuts or biases [49, 50]. However, in addition to the explainability of the ML model itself, for analytical performance evaluation of an AI application, explanations regarding the training dataset (including quantity, quality, uniqueness, annotation process, and scope and origin of the training data) are also needed to enable the identification of possible biases, gaps or shortcomings in the training‐data, which might cause the AI‐application to generate wrong results under real‐world conditions.

### Role of explainability and causability in clinical performance evaluation

The clinical performance of an AI application is evaluated in clinical studies where medical experts in diagnostics apply the AI application. This includes evaluating the human–AI interface for its ability to support medical decision‐making under real‐world conditions. To achieve high causability, the human–AI interface should provide the user (on request) with information about the AI application's scientific validation and performance evaluation, including comprehensible information about the algorithmic approach, the training and test datasets, as well as the reference dataset used for analytical performance evaluation. In addition, the human–AI interface shall also highlight relevant medical factors in the decision‐making of the AI application to enable the medical expert to understand why the AI application generated a specific result. The human–AI interface should provide explanatory information without disturbing or decelerating the user's primary task. It must ensure an effective mapping between explanations generated by an XAI method and the user's previous knowledge and consider the needs and preferences of the target user group.

## Conclusion and future outlook

Due to the large amounts of data available and increasing computing power, AI methods are becoming progressively successful in pathology and other fields. Currently, the most successful AI algorithms are based on the family of so‐called DL algorithms. These ML algorithms, which function based on neural networks, are called ‘black boxes’ because the obtained results are practically untraceable, and it remains – even to the AI expert – often unclear why an algorithm made a particular decision.

Traditionally, AI experts evaluate ML algorithms with metrics such as accuracy and specificity. These metrics are easily measurable on test data, and if the algorithm works as expected, this evaluation is also practically sufficient. However, these metrics can be dramatically misleading if, for example, the training data are not independent and identically distributed – which is rarely the case in clinical data. As a result, problems in the relations learned by the AI can remain hidden and lead to unexpected errors. Especially in medicine, when it comes to sensitive data and decisions that directly affect people, a better understanding is necessary to avoid damage and mistakes. To make these black boxes transparent, the field of XAI has developed a set of useful methods to highlight decision‐relevant parts that contributed to model accuracy in training or a particular prediction. However, a disadvantage is that these methods do not refer to a human model, i.e. a user.

For this reason, ‘causability’ was introduced in reference to the well‐known term usability. Whilst XAI means implementing transparency and traceability, causability is measuring the quality of explanations. Causability can thus be defined as ‘cause identifiability’ – the measurable extent to which an explanation of an AI's decision to a user achieves a specified level of causal understanding with effectiveness, efficiency, and satisfaction in a specified context of use.

In the future, novel human–AI interfaces that enable contextual understanding and allow the domain expert to ask interactive ‘what‐if’‐questions are urgently required to achieve a high level of causability. A human‐in‐the‐loop can (sometimes – not always) bring human experience and conceptual knowledge to AI processes – something that the current best AI algorithms (still) lack. Consequently, human medical professionals will remain important in the future and together with algorithms, will achieve better quality in their daily work. Digital pathology is a vital enabler in that direction.

## Author contributions statement

All authors contributed to the concept and writing of this review article and approved the submitted and published versions.
